# Plant performance was greater in the soils of more distantly related plants for an herbaceous understory species

**DOI:** 10.1093/aobpla/plx005

**Published:** 2017-02-07

**Authors:** Drake D. Sweet, Jean H. Burns

**Affiliations:** Department of Biology, Case Western Reserve University, 10900 Euclid Avenue, Cleveland, OH 44106, USA

**Keywords:** Abundance, community composition, phylogenetic signal, phylogeny, plant–soil feedbacks, soil mutualists, soil pathogens

## Abstract

Growing evidence suggests that plant–soil interactions have important implications for plant community composition. However, the role of phylogenetic relatedness in governing interactions between plants and soil biota is unclear, and more case studies are needed to help build a general picture of whether and how phylogeny might influence plant–soil interactions. We performed a glasshouse experiment to test whether degree of phylogenetic relatedness between *Aquilegia canadensis* and six co-occurring heterospecifics affects *A. canadensis* biomass through soil legacy effects. We also compared performance of *A. canadensis* in soils conditioned by invasive *Alliaria petiolata* versus native heterospecifics, hypothesizing that conditioning by *A. petiolata* would suppress the performance of the focal native plant. *A. canadensis* performed significantly better in distant relatives’ soils than in close relatives’ soils, and this effect disappeared with soil sterilization, consistent with close relatives sharing similar pathogens. Contrary to our expectations, soils conditioned by the invasive species *A. petiolata* versus by native species had similar effects on *A. canadensis*. The greater performance of *A. canadensis* in soils of more versus less distant relatives is consistent with a hypothesis of phylogenetically constrained pathogen escape, a phenomenon expected to promote coexistence of phylogenetically distant species. However, pairwise plant–soil feedback experiments are needed to create a stronger coexistence prediction.

## Teaser

We performed a glasshouse experiment to test whether degree of phylogenetic relatedness between Aquilegia canadensis and six co-occurring heterospecifics affects A. canadensis biomass through soil legacy effects. We found that A. canadensis performed significantly better in distant relatives' soils than in close relatives' soils, and this effect disappeared with soil sterilization. The greater performance of A. canadensis in soils of more versus less distant relatives is consistent with a hypothesis of phylogenetically-constrained pathogen escape, a phenomenon expected to promote coexistence of phylogenetically distant species.

## Introduction

Recent evidence suggests that plant community diversity is influenced by plant–soil feedbacks (PSFs) and soil legacy effects, where plants alter the performance of the next plant to grow in a given patch of soil (Kulmatiski *et al.* 2008; Bever *et al.* 2010, 2015). Plant-soil interactions are likely to be influenced by many factors, including: plant phylogeny, soil biota, and the native or introduced status of soil-conditioning plants. Here, we explore how phylogenetic relatedness influences the response of an herbaceous species, *Aquilegia canadensis*, to soil conditioning by heterospecifics. We include sterile soil controls to gain insight on the biotic drivers of plant-soil interactions (e.g. mutualists vs. pathogens), and compare the effects of native conditioning species with the invasive plant, *Alliaria petiolata*.

Because closely related species often share similar characteristics such as root morphology and root exudates (Comas and Eissenstat 2009; Jacquemyn *et al.* 2011; Martos *et al.* 2012; Reinhart *et al.* 2012; Valverde-Barrantes *et al.* 2014), and because close relatives might have similar soil microbial communities in their root zones (e.g. Burns *et al.* 2015), plant species might respond similarly to the soils of closely related plants. A recent meta-analysis found that phylogenetic distance was a weak predictor of relative performance in conspecific and heterospecific soils (Mehrabi and Tuck 2015). However, performance in conspecific and heterospecific soils (‘net whole soil feedback’) was more similar for close relatives across 57 old-field species, and the strength of the PSF correlated with abundance patterns in these species (Anacker *et al.* 2014). Case studies have also found conflicting evidence for a correlation between phylogeny and plant–soil interactions (Liu *et al.* 2012; Mehrabi *et al.* 2015; Miller and Menalled 2015; Münzbergová and Šurinová 2015).

Soil microbial communities influence plant performance through effects of both soil mutualists, like mycorrhizal fungi, and pathogens (Bever 2003; Brinkman *et al.* 2010; Diez *et al.* 2010; Kulmatiski *et al.* 2008). For example, oomycete pathogens build up in the soils of *Prunus serotina* and suppress plant performance (Packer and Clay 2000). However, the relative importance of mutualists and pathogens to plant performance is probably system specific, and has been poorly characterized for understory herbaceous plants (Kulmatiski *et al.* 2008).

Plant–soil interactions may also depend on the native or introduced status of the plants. Non-native, introduced plants often become invasive when they enter an area that lacks their natural enemies, thus allowing them to become highly abundant and reduce the success of native competitors (Callaway *et al.* 2004). Another mechanism for plant invasiveness is the production of allelochemicals that reduce native plant performance (‘novel weapons’) (Callaway and Ridenour 2004). In North American temperate forests, the invasive species *A. petiolata* (garlic mustard) is known to influence soil biota through allelochemicals (Roberts and Anderson 2001; Prati and Bossdorf 2004; Barto *et al.* 2011; Cantor *et al.* 2011), which inhibit the growth and germination of arbuscular mycorrhizal fungi (AMF), which many herbaceous species utilize (Wang and Qiu 2006).

Here, we ask if the phylogenetic distance between various conditioning species and subsequently grown *A. canadensis* affects *A. canadensis* performance in live and sterilized soils. This study provides the first test for an effect of phylogenetic distance on soil legacy effects in temperate understory plants that isolates the effects of soil biota. If soil pathogens are more important to plant performance than soil mutualists, we expect a positive influence of soil sterilization on plant performance (Bever 2003; Kulmatiski *et al.* 2008; Brinkman *et al.* 2010; Diez *et al.* 2010). Alternatively, because many of these native herbaceous species are dependent on AMF mutualists (Wang and Qiu 2006), positive effects of soil biota could lead to greater performance in live than sterilized soils. Because the invasive species *A. petiolata* is known to have allelopathic effects, we also predicted that it might suppress plant performance more than native species.

## Methods

This experiment took place during the summer of 2014 at Squire Valleevue Farm in Hunting Valley, OH, USA (41°29′ N, 81°25′ W). We chose *A. canadensis* as our focal species because it germinates readily in the lab and, like most spring flowering herbaceous understory species, it is a long-lived perennial that associates with AMF (Hale 2007). Conditioning species were selected based on their taxonomic distance from *A. canadensis*, with two confamilial species, two species in the same order, and two more distantly related species **[see ****Supporting Information—Table S1****].** All species were native, except the distantly related *A. petiolata*, which is introduced.

### Plant collection and soil conditioning

**Figure 1:**
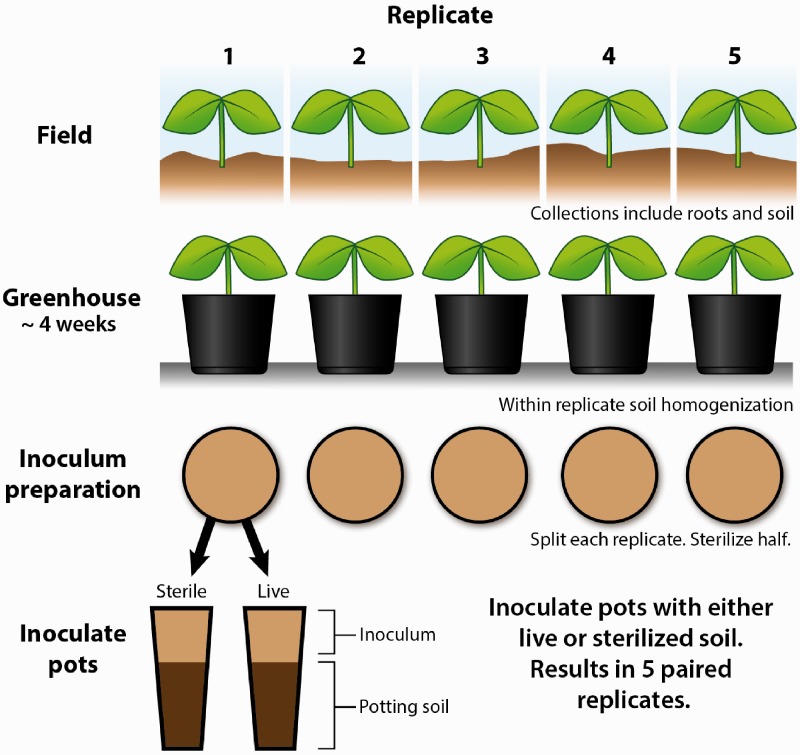
Experimental methods for conditioning soils. Five replicates of each of six heterospecific species **[see ****Supporting Information—Table S1****]** were used to condition a mix of potting and field soils (see ‘Plant Collection and Soil Conditioning’ section), conditioned soils were either left ‘live’ or ‘sterilized’, and then *A. canadensis* was grown in each experimental treatment, resulting in paired live and sterilized soil replicates. Figure credit: Catherine Stanley, www.stanleyillustration.com, stanleyillustration@gmail.com.

Five replicates of each plant species were identified and collected from the field, including soil from around the root zone of each species (Fig. 1). Whole, mature plants of roughly the same size and age (based on vegetation and perennial root-growth) were collected. Plants were collected at least 10 m from one another. After collection, plants were transplanted into pots containing a mixture of equal amounts of untreated potting soil and field soil from the root zone, keeping each species and replicate separate to accurately assess the variance across soil collections (Reinhart and Rinella 2016, Fig. 1). All conditioning plants were grown in 140 mm diameter (1.65 L) pots under a randomized placement on a potting bench inside the glasshouse and were watered by hand daily or as needed. Plants conditioned their soils for ∼4 weeks before their soil was collected for sterilization and inoculation.

### Inoculum preparation and sterilization

After the soil was conditioned, it was removed and homogenized using a standard US 2 mm testing sieve, again fully maintaining the independence of replicates. All tools/equipment were sterilized between each replicate collection using a stock solution of 10 % diluted bleach. Sterilized tools were also rinsed thoroughly in water to prevent residual bleach from making contact with live soil. Half of the soil was separated for sterilization (Fig. 1) and autoclaved using a standard laboratory autoclave (Market Forge Sterilematic, model STM-E) at 121 °C for 2 h, again, fully maintaining the independence of replicate collections. The result is a paired design, with sterile and live treatments within each conditioning replicate (Fig. 1).

### Focal pot preparation and inoculation

Deepots (6.4 cm diameter × 25.4 cm deep; Stuewe and Sons, Tangent, OR, USA) were prepared outside the glasshouse for planting *A. canadensis*. A meter-wide strip of weed cloth was laid along the north edge of the glasshouse and deepots were placed in blocks spaced out equally along its length. Position on the north side of the glasshouse reduced the average insolation the plants received in an attempt to mimic lower light conditions experienced in a forest understory. Pots were filled with untreated potting soil, watered, and allowed to settle naturally with no manual compression until they were ∼90 % full with roughly 500 mL of potting soil. Inoculation was achieved by adding 50 mL of conditioned soil on top of the potting soil directly prior to planting (Fig. 1). Equipment was sterilized between inoculations. Sterile and live soils were clustered within a block to avoid splash-contaminating sterilized soils during watering. Splash was minimized through spacing and careful watering techniques. Treatments were randomly assigned within blocks, and live and sterilized blocks were randomly assigned positions.

### Germination, planting and harvesting

The focal species *A. canadensis* was grown in both live and sterilized soil from each of six heterospecific conditioning species **[see ****Supporting Information—Table S1****]**, across five-paired replicates (Fig. 1). Seeds of the focal species were ordered online through Prairie Moon Nursery (Winona, MN), cold treated for ∼60 days at ∼2 ºC, germinated in a wet sand substrate, then placed into a growth chamber set for 12 h of light. Seedlings were planted into treatment pots, left outside of the glasshouse from August to October, and were brought inside the glasshouse to finish growing before the first frost of the season. Plants were watered by hand daily using a mister, or as needed, depending on weather conditions and apparent soil moisture. In December plants were separated into aboveground and belowground biomass, roots were washed and dried for 7 days in a drying oven set to 30 °C. Once dried, the plants were weighed in grams using a digital scale at a resolution of 0.0001 g.

### Phylogeny estimation

**Figure 2:**
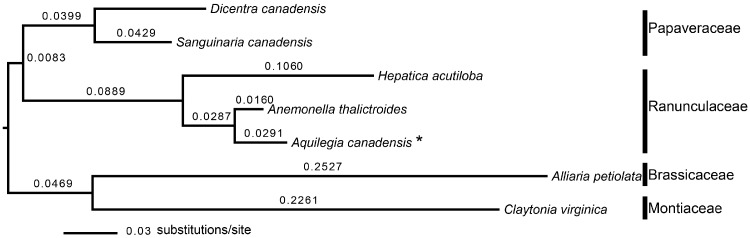
The molecular maximum likelihood phylogeny (phylogram), based on four DNA regions (*rbc*L, *mat*K, *trn*L-*trn*F, ITS, **[see ****Supporting Information—Table S1****]**), with branch lengths in substitutions/site above the branches. *Focal species *A. canadensis* was grown in the glasshouse-conditioned soils of six heterospecifics.

To estimate phylogenetic distances among species in the experiment, and to ensure that results were robust, we compared two estimated phylogenies. First, we used a molecular phylogeny, estimated *de novo* for taxa in this experiment **[see****Supporting Information—Table S1****]**. We aligned each DNA region separately in MUSCLE (Edgar *2004a, b*) in the MEGA platform (version 5.2.2) and concatenated four DNA regions for analysis **[see ****Supporting Information—Table S1****].** We conducted a maximum likelihood tree search in Garli (Zwickl 2006; version 0.951) and branch lengths are in substitutions per site (Fig. 2). The result is a phylogram with fine-scale variation in phylogenetic distances among species pairs. Second, we used phyloGenerator’s (Pearse and Purvis 2013) *congeneric.merge* function, which matches species in our sampled species list to genera on the global angiosperm phylogeny of Zanne *et al.* (2014). Because the genus *Anemonella* was not present in this phylogeny, we used the synonym *Anemone thalictoides*, in place of the now accepted name *Anemonella thalictroides* when conducting this merging function. The result is an ultrametric tree, with branch lengths in approximate millions of years **[see ****Supporting Information—Fig. S1**]. The phylogenetic distance between species was calculated as the summed branch lengths between them on the phylogenies. The placement of *Anemonella* in the Ranunculaceae was different for these two approaches, and the typology of these phylogenies was otherwise congruent (Figs 2)**[see****Supporting Information—Fig. S1**].

### Data analysis

Total biomass of *A. canadensis* was log-transformed for all analyses and we used generalized linear models (GLMs) with Gaussian error structure throughout. We tested for a block effect, and finding no evidence for an effect of block (P > 0.10), we excluded block from all analyses. We tested for a paired replicate (Fig. 1) effect, and finding no evidence for a pair effect (*P* > 0.25), we excluded pair from all analyses. All analyses were conducted in R (version 3.0.2) (R Development Core Team 2008). Residual plots were examined, and model assumptions were well-met. Data were archived at the Dryad Digital Repository (Sweet and Burns 2017).

To test for effects of phylogenetic distance on *A. canadensis* plant performance, we modelled total biomass as a function of phylogenetic distance to the conditioning plant, sterilization treatment, and the phylogenetic distance by sterilization interaction. We compared models with phylogenetic distances based on the molecular phylogeny with those from the phyloGenerator method. Because the molecular phylogeny resulted in a more continuous range of phylogenetic distances (Figs 2, **[see****Supporting Information—Table S1****]**), we present regressions with this measure of phylogenetic distances. Because we hypothesized that phylogenetic distance effects would differ in live and sterilized treatments (Liu *et al.* 2012), we used linear models within live and sterilized treatments to describe the strength of the correlation. To account for the non-independence in the data, because branch lengths on the phylogeny may be counted more than once, we used Mantel tests on distance matrices estimated using Euclidean distances (*sensu*Weiblen *et al.* 2006; Violle *et al.* 2011; Reinhart and Anacker 2014) to test for correlations between phylogenetic distance and plant total biomass. We also conducted Mantel tests with phylogenetic distance as a predictor with and without *A. petiolata* conditioned soils, to determine whether this invasive conditioning species influenced this relationship, compared with analyses including only the other five, native conditioning species.

To determine whether *A. canadensis* biomass depended on soil sterilization treatment or conditioning species, we used a GLM with sterilization treatment, conditioning species, and their interaction as predictors. To determine whether the introduced species, *A. petiolata*, had different effects than the native species, we contrasted native to introduced status of the conditioning species as an *a priori* contrast within this model and report the main effect and whether this contrast depended on sterilization treatment. 

## Results

**Figure 3:**
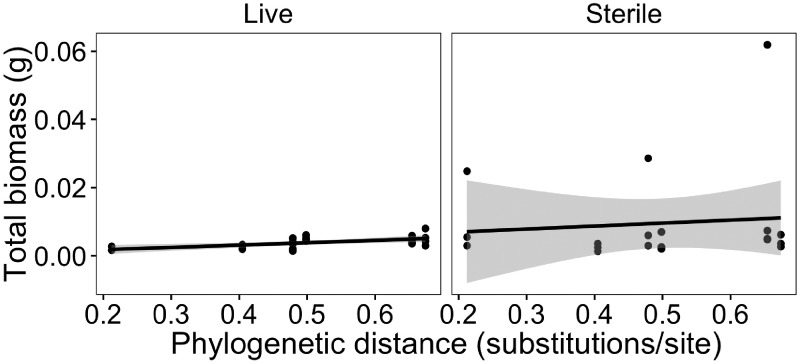
The biomass of *A. canadensis* was correlated with the phylogenetic distance to heterospecific conditioning species in live field soils but not in sterilized soils.

*A. canadensis* plant performance was greater in soils conditioned by more distant relatives, both when phylogenetic distances were based on a molecular phylogram (*P* = 0.09, Fig. 2, Table 1) or on a phyloGenerator ultrametric phylogeny (*P* = 0.04, Table 1). The slopes for this relationship did not depend on soil sterilization treatment (Table 1); however, phylogeny was a stronger predictor of plant biomass in live than sterilized soils, according to Mantel tests that take non-independence of branch lengths into account. Plant total biomass was greater for more phylogenetically distant heterospecific conditioning species in live soils (Fig. 3; Mantel test: Pearson correlation coefficient *r* = 0.24, *P* = 0.012). This relationship was no longer significant in sterilized soils (Fig. 3; *r* = −0.03, *P* = 0.60). When the invasive conditioning species *A. petiolata* was excluded from these Mantel tests, phylogenetic distance still correlated positively with *A. canadensis* biomass in live soils (Pearson correlation coefficient *r* = 0.18, *P* = 0.047) and again did not correlate with *A. canadensis* biomass in sterilized soils (Pearson correlation coefficient *r* = 0.03, *P* = 0.36).

**Table 1.** Total biomass of *A. canadensis* as a function of soil sterilization treatment, phylogenetic distance between the focal species and the conditioning species, and the sterilization by phylogenetic distance interaction.

Predictor	df	Deviance	Residual deviance	F-ratio	P-value
**molecular phylogeny**					
Soil sterilization treatment	1,39	1.89	21.84	3.53	0.07
Phylogenetic Distance (substitutions/site)	1,38	1.61	20.23	3.02	0.09
Sterilization × Phylogenetic Distance	1,37	0.41	19.81	0.77	0.39
**phyloGenerator phylogeny**					
Soil sterilization treatment	1,39	1.89	21.84	3.61	0.07
**Phylogenetic Distance (MYA)**	**1,38**	**2.33**	**19.52**	**4.44**	**0.04**
Sterilization × Phylogenetic Distance	1,37	0.15	19.37	0.28	0.60

**Figure 4:**
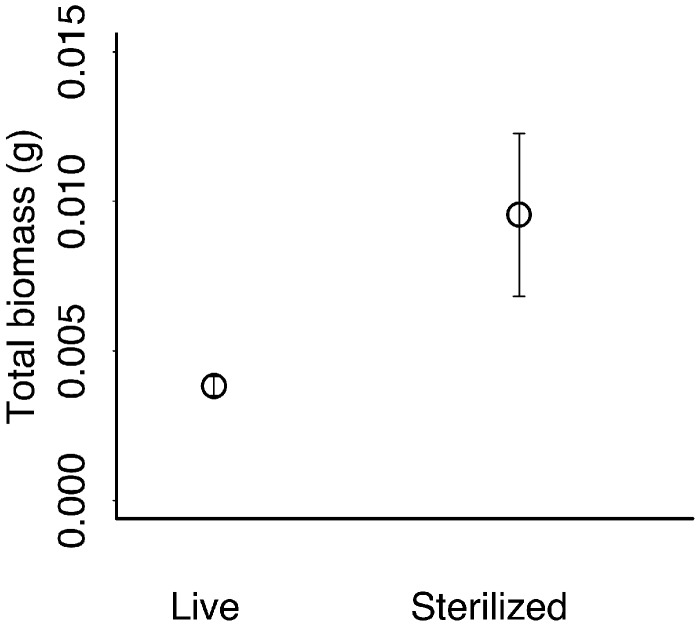
The biomass of *A. canadensis* across soil sterilization treatments (Table 1).

*A. canadensis* plant biomass was greater in sterilized than live soils (Fig. 4) and sterilization did not interact with conditioning species identity **[see****Supporting Information—Table S2****]***A. petiolata* did not have a different influence on *A. canadensis* plant biomass, compared with native conditioning species (*t*_1,34_ = 1.14, *P* = 0.26), nor did the effect of native status depend on sterilization treatment (t_1,29_ = −1.31, *P* = 0.20).

## Discussion

*A. canadensis* performed better in soils conditioned by more distantly related species, consistent with our hypothesis that closely related species inhibit one another through phylogenetically conserved interactions with soil microbiota, a finding consistent with results in other study systems (Liu *et al.* 2012). Second, we found that soil sterilization increased plant performance in *A. canadensis*, confirming our hypothesis that soil biota are inhibitory for this herbaceous species and suggesting that soil pathogens may play a larger role than AMF mutualists (Bever 2003; Kulmatiski *et al.* 2008; Diez *et al.* 2010). Contrary to our predictions, soil conditioned by the invasive species *A. petiolata* did not reduce performance of *A. canadensis* more than soils conditioned by native species, consistent with some evidence that *A. petiolata* may be experiencing selection for reduced allelopathy at high population densities (Lankau *et al.* 2009).

Our results are consistent with some (Liu *et al.* 2012), but not all (e.g. Mehrabi and Tuck 2015) studies of phylogenetic effects on plant–soil interactions. Many factors could explain variation in the relationship between plant phylogeny and plant–soil interactions. Experimental methods for studying PSFs vary greatly, and include field collected soils, glasshouse conditioned soils, and considerable variation in the length of time plants condition the soil, among other factors (Brinkman *et al.* 2010), and studies that find different patterns also frequently used different methods (cf. Liu *et al.* 2012; Mehrabi and Tuck 2015). Some of these apparent discrepancies could also be influenced by variation in phylogenetic scale among studies. For example, Mehrabi and Tuck (2015) studied soil conditioning at the level of plant families, and found no correlation between phylogenetic distance and the strength of PSFs. Alternatively, Liu *et al.*’s (2012) comparison included congeners and confamilials, as have other studies finding that congeners similarly influence one another through the soil (Diez *et al.* 2010; Burns and Strauss 2011; Callaway *et al.* 2013). Because our study includes two confamilials, but no congeners, it is intermediate in phylogenetic scale, relative to other studies. Future studies should consider manipulating experimental method (e.g. length of conditioning) and phylogenetic scale to directly address these issues. The relative importance of mutualists, pathogens, and other drivers of PSFs are also likely to vary among study systems, and comparisons across ecological gradients are still needed (Smith and Reynolds 2015).

Our comparison of live with sterilized soils suggests that a role of phylogeny could be driven in part by soil biota for *A. canadensis* (Bever *et al.* 2010; Brinkman *et al.* 2010). This result is consistent with a previous study in a tropical tree, where *Castanopsis fissa* had greater survival in soils of more distant relatives in live soils and not fungicide-treated soils (Liu *et al.* 2012). Thus fungal pathogens in the soil might be shared by closely related plant species, and escape from these pathogens might increase plant performance. Closely related plants have also been shown to have similar soil fungal communities in their root zones in the field, even for congeners that occur in different habitat types (Burns *et al.* 2015), also consistent with plant relatedness influencing soil microbiota. Studies of foliar pathogens also suggest that phylogeny might influence how microbes structure plant communities (Parker *et al.* 2015), suggesting that phylogenetic conservation of pathogen effects, both above and below ground, might have broad implications for plant community structure.

Our evidence is consistent with pathogens influencing performance in heterospecific soils (Bever *et al.* 2015); however, an alternate explanation for greater performance in sterilized soils is that mycorrhizal fungi can be parasitic if the net cost of symbioses is greater than the net benefit received by the host plant (Johnson *et al.* 1997; Lapointe and Molard 1997). AMF fungi can function along a continuum ranging from mutualistic to parasitic, depending on environmental conditions and the needs of the host plant (Johnson 1993; Klironomos 2003). In the stressful conditions that plants often experience in the field, AMF mutualists can be highly beneficial, but under less stressful conditions such as those experienced in a glasshouse setting, the cost of harboring AMF fungi may actually be greater than the benefits received. This could be an alternative explanation for why we observed greater *A. canadensis* biomass in sterilized compared with live heterospecific soils.

Due to the large amount of literature documenting the negative allelopathic effects of *A. petiolata* on native plant communities (e.g. Roberts and Anderson 2001; Prati and Bossdorf 2004; Barto *et al.* 2011; Cantor *et al.* 2011), we expected to find that soil conditioned by *A. petiolata* would decrease plant performance in a native plant, *A. canadensis*. However, our results showed effects of *A. petiolata* conditioned soil did not differ from effects of native species, on average. This was surprising considering *A. petiolata* limits the growth of AMF (Cantor *et al.* 2011), which *A. canadensis* utilizes (Wang and Qiu 2006). One explanation for this finding may be that there are differences in allelopathic concentration in aboveground and belowground structures in *A. petiolata*, suggesting that allelochemical deposition in the soil may be more significant coming from senescing leaves and stems (Cantor *et al.* 2011), rather than the roots which we used to condition the soil. Because *A. petiolata* is an invasive species that spreads rapidly throughout an invaded area, this species often forms large monocultures. Populations of this species that had been established for 50 years showed reduced production of allelopathic chemicals (Lankau et al. 2009), perhaps because of the inhibitory effect *A. petiolata* has on its own species at high densities (Pardini *et al.* 2009, 2011), which should select for reduced allelopathy at older invasion sites. The specimens collected from the field were part of a high-density patch of *A. petiolata*. Because the field site for this experiment has been invaded for a long period of time, this is a reasonable explanation for why garlic mustard was not found to inhibit the performance of *A. canadensis*.

## Conclusions and Future Directions

If plants often perform better in the soils of more distant relatives, plant–soil interactions could explain patterns in community composition, such as the frequent observation of greater co-occurrence between distantly related plants at small spatial scales (Valiente-Banuet and Verdu 2007; Vamosi *et al.* 2009). A common explanation for this pattern is competition: if closely related plants are similar in their niche (Darwin 1859), they might compete more strongly than more distant relatives (e.g. Burns and Strauss 2011). Thus competitive exclusion by close relatives could lead to communities of more distantly related species (Webb *et al.* 2002). Our results suggest that plant–soil interactions could be an alternative explanation in some systems. If plants escape from pathogens in distant relatives’ soils, plant communities should be composed of more distantly related species than a random assemblage (Liu *et al.* 2012). This effect has been called a ‘phylogenetic Janzen-Connell’ effect (Liu *et al.* 2012), and could help explain the high diversity in systems such as tropical trees (Liu *et al.* 2012) and perhaps temperate herbaceous understories. Future research employing pairwise PSF experiments with con- and heterospecific plants of varying phylogenetic relatedness is needed to fully test the phylogenetic Janzen-Connell effect in temperate woodland and other systems.

## Sources of Funding

This work was funded by the National Science Foundation (DEB 1250170); D.D.S. was supported by an REU supplement to this award.

## Contributions by the Authors

J.H.B. conceived the idea. J.H.B. and D.D.S. designed and performed the experiment, analysed the data, and wrote the manuscript.

## Conflicts of Interest Statement

None declared.

## Supplementary Material

Supplementary Data

## References

[plx005-B1] Anacker BL Klironomos JN Maherali H Reinhart KO Strauss SY. 2014 Phylogenetic conservatism in plant-soil feedback and its implications for plant abundance. Ecology Letters, 17:1613–1621. 25328022 10.1111/ele.12378

[plx005-B2] Barto EK Antunes PM Stinson K Koch AM Klironomos JN Cipollini D. 2011 Differences in arbuscular mycorrhizal fungal communities associated with sugar maple seedlings in and outside of invaded garlic mustard forest patches. Biological Invasions, 13:2755–2762.

[plx005-B3] Bever JD. 2003 Soil community feedback and the coexistence of competitors: conceptual frameworks and empirical tests. New Phytologist, 157:465–473. 10.1046/j.1469-8137.2003.00714.x 33873396

[plx005-B4] Bever JD Dickie IA Facelli E Facelli JM Klironomos J Moora M Rillig MC Stock WD Tibbett M Zobel M. 2010 Rooting theories of plant community ecology in microbial interactions. Trends in Ecology and Evolution, 25:468–478. 20557974 10.1016/j.tree.2010.05.004 PMC2921684

[plx005-B5] Bever JD Mangan SA Alexander HM. 2015 Maintenance of Plant Species Diversity by Pathogens. Annual Review of Ecology, Evolution, and Systematics, 46:305–325.

[plx005-B6] Brinkman E Putten W Bakker E Verhoeven K. 2010 Plant-soil feedback: Experimental approaches, statistical analyses and ecological interpretations. Journal of Ecology, 98:1063–1073.

[plx005-B7] Burns JH Anacker BL Strauss SY Burke DJ. 2015 Soil microbial community variation correlates most strongly with plant species identity, followed by soil chemistry, spatial location and plant genus. AoB PLANTS 7:plv030. 25818073 10.1093/aobpla/plv030 PMC4417136

[plx005-B8] Burns JH Strauss SY. 2011 More closely related species are more ecologically similar in an experimental test. Proceedings of the National Academy of Sciences of the United States of America 108:5302–5307. 21402914 10.1073/pnas.1013003108 PMC3069184

[plx005-B9] Callaway RM Montesinos D Williams K Maron JL. 2013 Native congeners provide biotic resistance to invasive *Potentilla* through soil biota. Ecology 94:1223–1229. 23923481 10.1890/12-1875.1

[plx005-B10] Callaway RM Thelen GC Rodriguez A Holben WE. 2004 Soil biota and exotic plant invasion. Nature 427:731–733. 14973484 10.1038/nature02322

[plx005-B11] Callaway RM Ridenour WM. 2004 Novel weapons: invasive success and the evolution of increase competitive ability. Frontiers in Ecology and the Environment 2:436–443.

[plx005-B12] Cantor A Hale A Aaron J Traw MB Kalisz S. 2011 Low allelochemical concentrations detected in garlic mustard-invaded forest soils inhibit fungal growth and AMF spore germination. Biological Invasions 13:3015–3025.

[plx005-B13] Comas LH Eissenstat DM. 2009 Patterns in root trait variation among 25 co‐existing North American forest species. New Phytologist 182:919–928. 19383105 10.1111/j.1469-8137.2009.02799.x

[plx005-B14] Darwin C. 1859 On the Origin of species. 1st edn London, UK: John Murray.

[plx005-B15] Diez J Dickie I Edwards G Hulme P Sullivan J Duncan R. 2010 Negative soil feedbacks accumulate over time for non-native plant species. Ecology Letters 13:803–809. 20482584 10.1111/j.1461-0248.2010.01474.x

[plx005-B16] Edgar RC. 2004a MUSCLE: a multiple sequence alignment method with reduced time and space complexity. Bmc Bioinformatics 5:1–19. 15318951 10.1186/1471-2105-5-113 PMC517706

[plx005-B17] Edgar RC. 2004b MUSCLE: multiple sequence alignment with high accuracy and high throughput. Nucleic Acids Research 32:1792–1797. 15034147 10.1093/nar/gkh340 PMC390337

[plx005-B18] Hale AN. 2007 An empirical test of the mutualism disruption hypothesis: impacts of an allelopathic invader on the ecophysiology of a native forest herb. PhD Dissertation, University of Pittsburgh.

[plx005-B19] Jacquemyn H Merckx V Brys R Tyteca D Cammue BPA Honnay O Lievens B. 2011 Analysis of network architecture reveals phylogenetic constraints on mycorrhizal specificity in the genus Orchis (Orchidaceae). New Phytologist 192:518–528. 21668874 10.1111/j.1469-8137.2011.03796.x

[plx005-B20] Johnson NC. 1993 Can fertilization of soil select less mutualistic mycorrhizae?. Ecological Applications 3:749–757. 27759303 10.2307/1942106

[plx005-B21] Johnson NC Graham JH Smith FA. 1997 Functioning of mycorrhizal associations along the mutualism–parasitism continuum. New Phytologist 135:575–585.

[plx005-B22] Klironomos JN. 2003 Variation in plant response to native and exotic arbuscular mycorrhizal fungi. Ecology 84:2292–2301.

[plx005-B23] Kulmatiski A Beard K Stevens J Cobbold S. 2008 Plant-soil feedbacks: a meta-analytical review. Ecology Letters 11:980–992. 18522641 10.1111/j.1461-0248.2008.01209.x

[plx005-B24] Lankau RA Nuzzo V Spyreas G Davis AS. 2009 Evolutionary limits ameliorate the negative impact of an invasive plant. Proceedings of the National Academy of Sciences of the United States of America 106:15362–15367. 19706431 10.1073/pnas.0905446106 PMC2730356

[plx005-B25] Lapointe L Molard J. 1997 Costs and benefits of mycorrhizal infection in a spring ephemeral, *Erythronium americanum*. New Phytologist 135:491–500.

[plx005-B26] Liu X Liang M Etienne RS Wang Y Staehelin D Yu S. 2012 Experimental evidence for a phylogenetic Janzen-Connell effect in a subtropical forest. Ecology Letters 15:111–118. 22082078 10.1111/j.1461-0248.2011.01715.x

[plx005-B27] Martos F Munoz F Pailler T Kottke I Gonneau C Selosse MA. 2012 The role of epiphytism in architecture and evolutionary constraint within mycorrhizal networks of tropical orchids. Molecular Ecology 21:5098–5109. 22765763 10.1111/j.1365-294X.2012.05692.x

[plx005-B28] Mehrabi Z Bell T Lewis OT. 2015 Plant-soil feedbacks from 30-year family-specific soil cultures: phylogeny, soil chemistry and plant life stage. Ecological Evolution 5:2333–2339. 10.1002/ece3.1487 PMC4475366 26120423

[plx005-B29] Mehrabi Z Tuck SL. 2015 Relatedness is a poor predictor of negative plant–soil feedbacks. New Phytologist 205:1071–1075. 25557183 10.1111/nph.13238 PMC4303931

[plx005-B30] Miller ZJ Menalled FD. 2015 Impact of species identity and phylogenetic relatedness on biologically-mediated plant-soil feedbacks in a low and a high intensity agroecosystem. Plant Soil 389:171–183.

[plx005-B31] Münzbergová M Šurinová M. 2015 The importance of species phylogenetic relationships and species traits for the intensity of plant-soil feedback. Ecosphere 6:234.

[plx005-B32] Packer A Clay K. 2000 Soil pathogens and spatial patterns of seedling mortality in a temperate tree. Nature 404:278–281. 10749209 10.1038/35005072

[plx005-B33] Pardini EA Drake JM Chase JM Knight TM. 2009 Complex population dynamics and control of the invasive biennial *Alliaria petiolata* (garlic mustard). Ecological Applications 19:387–397. 19323197 10.1890/08-0845.1

[plx005-B34] Pardini EA Drake JM Knight TM. 2011 On the utility of population models for invasive plant management: response to Evans and Davis. Ecological Applications 21:614–618.

[plx005-B35] Parker IM Saunders M Bontrager M Weitz AP Hendricks R Magarey R Suiter K Gilbert GS. 2015 Phylogenetic structure and host abundance drive disease pressure in communities. Nature 520:542–546. 25903634 10.1038/nature14372

[plx005-B36] Pearse WD Purvis A. 2013 phyloGenerator: an automated phylogeny generation tool for ecologists. Methods in Ecology and Evolution 4:692–698.

[plx005-B37] Prati D Bossdorf O. 2004 Allelopathic inhibition of germination by *Alliaria petiolata* (Brassicaceae). American Journal of Botany 91:285–288. 21653384 10.3732/ajb.91.2.285

[plx005-B38] R Development Core Team. 2008 R: A Language and Environment for Statistical Computing. Vienna, Austria: R Foundation for Statistical Computing http://www.R-project.org/ (23 January 2017).

[plx005-B39] Reinhart KO Anacker BL. 2014 More closely related plants have more distinct mycorrhizal communities. AoB Plants 6:plu051. 25165062 10.1093/aobpla/plu051 PMC4172195

[plx005-B40] Reinhart KO Rinella MJ. 2016 A common soil handling technique can generate incorrect estimates of soil biota effects on plants. New Phytologist 210:786–789. 26738893 10.1111/nph.13822

[plx005-B41] Reinhart KO Wilson GW Rinella MJ. 2012 Predicting plant responses to mycorrhizae: integrating evolutionary history and plant traits. Ecology Letters 15:689–695. 22507627 10.1111/j.1461-0248.2012.01786.x

[plx005-B42] Roberts KJ Anderson RC. 2001 Effect of garlic mustard extracts on plants and arbuscular mycorrhizal (AM) fungi. American Midland Naturalist 146:146–152.

[plx005-B43] Smith LM Reynolds HL. 2015 Plant–soil feedbacks shift from negative to positive with decreasing light in forest understory species. Ecology 96:2523–2532. 26594708 10.1890/14-2150.1

[plx005-B44] Sweet DD Burns JH. 2017 Data from: Plant performance was greater in the soils of more distantly related plants for an herbaceous understory species. Dryad Digital Repository doi:10.5061/dryad.6528d. 10.1093/aobpla/plx005 PMC5499765 28702162

[plx005-B45] Valiente-Banuet A Verdu M. 2007 Facilitation can increase the phylogenetic diversity of plant communities. Ecology Letters 10:1029–1036. 17714492 10.1111/j.1461-0248.2007.01100.x

[plx005-B46] Valverde-Barrantes OJ Smemo KA Blackwood CB. 2014 Fine root morphology is phylogenetically structured, but nitrogen is related to the plant economics spectrum in temperate trees. Functional Ecology 29:796–807.

[plx005-B47] Vamosi SM Heard SB Vamosi JC Webb CO. 2009 Emerging patterns in the comparative analysis of phylogenetic community structure. Molecular Ecology 18:572–592. 19037898 10.1111/j.1365-294X.2008.04001.x

[plx005-B48] Violle C Nemergut DR Pu Z Jiang L. 2011 Phylogenetic limiting similarity and competitive exclusion. Ecology Letters 14:782–787. 21672121 10.1111/j.1461-0248.2011.01644.x

[plx005-B49] Wang B Qiu YL. 2006 Phylogenetic distribution and evolution of mycorrhizas in land plants. Mycorrhiza 16:299–363. 16845554 10.1007/s00572-005-0033-6

[plx005-B50] Webb CO Ackerly DD McPeek MA Donoghue MJ. 2002 Phylogenies and community ecology. Annual Review of Ecology and Systematics 33:475–505.

[plx005-B51] Weiblen GD Webb CO Novotny V Basset Y Miller SE. 2006 Phylogenetic dispersion of host use in a tropical insect herbivore community. Ecology 87:S62–S75. 16922303 10.1890/0012-9658(2006)87[62:pdohui]2.0.co;2

[plx005-B52] Zanne AE Tank DC Cornwell WK Eastman JM Smith SA FitzJohn RG McGlinn DJ O'Meara BC Moles AT Reich PB Royer DL Soltis DE Stevens PF Westoby M Wright IJ Aarssen L Bertin RI Calaminus A Govaerts R Hemmings F Leishman MR Oleksyn J Soltis PS Swenson NG Warman L Beaulieu JM. 2014 Three keys to the radiation of angiosperms into freezing environments. Nature 506:89–92. 24362564 10.1038/nature12872

[plx005-B53] Zwickl DJ. 2006 Genetic algorithm approaches for the phylogenetic analysis of large biological sequence datasets under the maximum likelihood criterion. PhD Dissertation, University of Texas, Austin, TX.

