# Supplementary material for: Plant performance was greater in the soils of more distantly related plants for an herbaceous understory species
Source: AoB Plants. 2017 Feb 7;9(1):plx005. doi: 10.1093/aobpla/plx005 (PMC5499765; doi:10.1093/aobpla/plx005)
Supplement: Supplementary Data [file plx005_Supp.docx]

**Table S1.** GenBank accession numbers used to estimate the phylogeny amongst sampled taxa.

| Species | Family | Taxonomic category* | *rbc*L | *mat*K | *trn*L-*trn*F | ITS |
| --- | --- | --- | --- | --- | --- | --- |
| *Alliaria petiolata* | Brassicaceae | Distant | JQ933212.1 | AF144363.1 | JN189781.1 | AF336218.1 |
| *Anemonella thalictroides* | Ranunculaceae | Confamilial | EU053924.1 | NA | JX573511.1 | JX233748.1 |
| *Aquilegia canadensis* | Ranunculaceae | Conspecific | AY392755.1 | EU827653.1 | NA | U75656.1 |
| *Claytonia virginica* | Montiaceae | Distant | NA | AY764113.1 | NA | AY764067.1 |
| *Dicentra canadensis* | Papaveraceae | Order | HQ590062.1 | HQ593267.1 | NA | AY198273.1 |
| *Hepatica acutiloba* | Ranunculaceae | Confamilial | HQ589952.1 | DQ994677.1 | AM268056.1 | AM267285.1 |
| *Sanguinaria canadensis* | Papaveraceae | Order | L01951.2 | DQ401350.1 | NA | GU983876.1 |

*Distance from the focal plant, *Aquilegia canadensis*

**Figure S1.** The ultrametric phyloGenerator phylogeny, with branch lengths in approximate millions of years. Branch lengths are shown above the branches.

Table S2. Total biomass of *Aquilegia canadensis* as a function of soil sterilization treatment, the conditioning species identity, and the sterilization by conditioning species interaction.

| Predictor | DF | Deviance | Residual Deviance | F-ratio | *P*-value |
| --- | --- | --- | --- | --- | --- |
| **Soil sterilization treatment** | **1,39** | **1.89** | **21.84** | **4.09** | **0.05** |
| Conditioning species | 5,34 | 3.97 | 17.88 | 1.71 | 0.16 |
| Sterilization × Conditioning species | 5,29 | 4.45 | 13.42 | 1.93 | 0.12 |
